# Immunomodulatory Therapy for Giant Cell Myocarditis: A Narrative Review

**DOI:** 10.7759/cureus.40439

**Published:** 2023-06-14

**Authors:** Muhammad Wahdan Naseeb, Victor O Adedara, Muhammad Talha Haseeb, Hareem Fatima, Swapna Gangasani, Kamaljit R Kailey, Moiz Ahmed, Kiran Abbas, Waleed Razzaq, Muhammad M Qayyom, Zain U Abdin

**Affiliations:** 1 Internal Medicine, Dow University of Health Sciences, Karachi, PAK; 2 Medicine, St. George's University School of Medicine, St. George's, GRD; 3 Internal Medicine, Shaikh Zayed Hospital, Lahore, PAK; 4 Internal Medicine, Federal Medical College, Islamabad, PAK; 5 Internal Medicine, New York Medical College (NYMC) St. Mary’s General Hospital and Saint Clare’s Hospitals, New Jersey, USA; 6 Medicine and Surgery, Gian Sagar Medical College and Hospital, Patiala, IND; 7 Cardiology, National Institute of Cardiovascular Diseases, Karachi, PAK; 8 Community Health Sciences, Aga Khan University, Karachi, PAK; 9 Internal Medicine, Services Hospital, Lahore, PAK; 10 Cardiology Department, ChristianaCare, Newark, USA; 11 Medicine, District Headquarter Hospital, Faisalabad, PAK

**Keywords:** myocarditis, intravenous igiv, immunomodulatory, immunosuppressive therapy, giant cell myocarditis, cyclosporin, azathioprine

## Abstract

Giant cell myocarditis (GCM) is a rare, often rapidly progressive, and potentially fatal disease because of myocardium inflammation due to the infiltration of giant cells triggered by infectious as well as non-infectious etiologies. Several studies have reported that GCM can occur in patients of all ages but is more commonly found in adults. It is relatively more common among African American and Hispanic patients than in the White population. Early diagnosis and treatment are critical. Electrocardiogram (EKG), complete blood count, erythrocyte sedimentation rate, C-reactive protein, and cardiac biomarkers such as troponin and brain natriuretic peptide (BNP), echocardiogram, cardiac magnetic resonance imaging (MRI), myocardial biopsy, and myocardial gene profiling are useful diagnostic tools. Current research has identified several potential biomarkers for GCM, including myocarditis-associated immune cells, cytokines, and other chemicals. The standard of care for GCM includes aggressive immunosuppressive therapy with corticosteroids and immunomodulatory agents like rituximab, cyclosporine, and infliximab, which have shown promising results in GCM by balancing the immune system and preventing the attack on healthy tissues, resulting in the reduction of inflammation, promotion of healing, and decreasing the necessity for cardiac transplantation. Without immunosuppression, the chance of mortality or cardiac surgery was 100%. Multiple studies have revealed that a treatment combination of corticosteroids and immunomodulatory agents is superior to corticosteroids alone. Combination therapy significantly increased transplant-free survival (TFS) and decreased the likelihood of heart transplantation, hence improving overall survival. It is important to balance the benefits of immunosuppression with its potentially adverse effects. In conclusion, immunomodulatory therapy adds significant long-term survival benefits to GCM.

## Introduction and background

Giant cell myocarditis (GCM) is a rare, rapidly progressing, and possibly fatal condition caused by inflammation of the myocardium with the invasion of giant cells, which can be caused by both infectious and non-infectious etiologies. This leads to detrimental consequences for the heart muscles and puts patients at significant risk for heart failure or cardiac arrest. Early recognition of the disease is crucial because it is frequently diagnosed at a late stage, which subsequently restricts the potential for recovery.

Patients typically experience symptoms such as arrhythmias or heart failure. Conventional treatments for GCM include immunosuppressive therapy and heart transplant, but have not provided a successful ratio of improving patient outcomes [1].

Recently, research has shown promising results with the use of immunomodulatory therapy, which has become a new and promising treatment option for people with GCM. Unlike immunosuppression, immunomodulatory therapy aims to balance the immune system and prevent it from attacking healthy tissues, which is the underlying cause of GCM. This therapy aims to reduce inflammation and promote healing in the myocardium. Some GCM patients have shown improved heart function and a decreased requirement for heart transplantation when receiving immunomodulatory therapy with drugs such as rituximab, cyclosporine, and infliximab [2]. Therefore, early diagnosis and treatment with immunomodulatory therapy are critical for improving patient outcomes.

The epidemiology of GCM is not well defined as it is considered a rare disease with an unknown incidence and prevalence. Globally, the prevalence of GCM has been calculated to be less than one case per million people, with an incidence of 0.1 to 1 case per million people annually [3-5]. GCM affects individuals of all ages equally, with an average onset in the fifth decade of life. Myocarditis, a significant cause of heart disease resulting from heart muscle inflammation, is estimated to have an annual incidence of around 22 to 66 cases per million people worldwide [5]. Evidence suggests that myocarditis primarily affects children and young adults, with the most affected age group being youngsters aged between 10 and 19 years [3,4].

According to a study in Africa, myocarditis is very common in those with acute heart failure [5]. Similarly, studies from South America and Asia have also reported higher rates of myocarditis in their respective regions [4,5]. In terms of geographical disparity, there is evidence of variation in the incidence of myocarditis globally, with higher rates observed in low- and middle-income countries [5]. Evidence suggests that the prevalence of myocarditis ranges from 0.5% to 5% among patients who undergo endomyocardial biopsy [4].

## Review

Epidemiology of GCM 

GCM is a rare disease that has been reported in patients of all ages and from different parts of the world [6]. A study reported that although it may occur in any part and any region of the world, its highest prevalence was observed in countries with low income as compared to those that are developed and have high income sources [6]. The income disparity is reflected in the fact that a study conducted in the United States found an incidence of GCM of 0.09 cases per million people per year [6], while a study in Japan reported an incidence of 0.08 cases per million people per year. When compared to countries with low incomes such as India, where GCM is extremely rare, the incidence rates are comparatively higher [7,8].

In general, GCM is more prevalent among young and middle-aged adults, with some studies indicating a slight male predominance. It has been reported in both pediatric and adult populations, with a median onset age in the fifth decade of life [3]. A study found that the median age of onset of myocarditis was 42 years; however, it can occur between the ages of five and 89 years [4]. The incidence of myocarditis peaked between the ages of 20 and 29 and then declined as people aged [8,9]. Evidence suggests that younger patients may experience more severe myocarditis, with a higher risk of acute heart failure and a poorer prognosis. Further studies have reported similar findings, indicating that although GCM can occur in patients of all ages, it is more commonly found in adults. According to a study, individuals with GCM ranged in age from 18 to 75 years, with the median age being 51 [9]. Another study reported that the average age of GCM patients was 44.5 years old, with 31% of those patients being under the age of 35 [10].

The GCM displays a disparity in several factors, including race and ethnicity. A study revealed that myocarditis was more prevalent among African American and Hispanic patients compared to White patients, with an odds ratio of 2.1 (95% confidence interval (CI), 1.4-3.2) for African Americans and 2.2 (95% CI, 1.4-3.4) for Hispanics after adjusting for age and sex [4]. This suggests that race and ethnicity play a crucial role in the development of myocarditis. Regarding the distribution among sexes, one study found that out of 383 patients with acute myocarditis, 67.8% were male and 32.2% were female [11]. Similarly, another study reported a male predominance in patients with biopsy-proven myocarditis, with a male-to-female ratio of 2.4:1 [12].

Pathogenesis 

Myocarditis refers to inflammation of myocardial tissue, which may occur in isolation or as part of systemic conditions. It can be classified as infectious or noninfectious. GCM comes under the noninfectious cause of myocarditis [13].

It is an often deadly disorder of cardiac myocytes that leads to their extensive degeneration and necrosis and is caused by immune dysregulation mediated by T lymphocytes [14-16]. Early infiltrated CD-4-positive T cells produce inflammatory cytokines such as interleukin-2 and interferon-gamma, causing the formation of giant cells characteristic of the disease. In later stages, the Th2 response may dominate, leading to the fibrosis of affected areas. The eosinophils in the infiltrate also increase cytokine production and release cytotoxic substances from their granules, like major basic proteins and eosinophil peroxidase [17].

The resultant effect of this T cell-mediated inflammation is the presence of a diffuse inflammatory infiltrate consisting of lymphocytes, macrophage-driven multinucleated giant cells, scattered plasma cells, and eosinophils in the myocardium [8,9]. In the acute phase, these inflammatory cells surround and directly attack the myocytes, causing extensive damage at multiple sites. As the disease progresses to a more chronic phase, inflammation becomes less diffuse, and reparative fibrosis starts [13].

Although the precise mechanism by which these immune cells cause myocyte destruction remains unclear, the association of GCM with other systemic autoimmune processes like inflammatory bowel disease, fibromyalgia, and Hashimoto's thyroiditis emphasizes the significance of autoimmunity in the pathogenesis of the disease [18,19]. Cases of GCM have been reported after the use of checkpoint inhibitor immunotherapy given for the treatment of metastatic melanoma and after allogeneic hematopoietic stem cell transplants, making its etiology a complex multifactorial process involving genetics and autoimmunity [18,20,21].

Clinical features 

GCM occurs primarily in previously healthy adults [15]. The usual clinical manifestation involves a swift progression of cardiac deterioration, resulting in cardiogenic shock [22]. Other early manifestations of the disease include bradyarrhythmias, supraventricular and ventricular tachyarrhythmias, and cardiac conduction abnormalities, including complete atrioventricular (AV) block. GCM may also take a more indolent course, leading to chronic heart failure. Rarely, it may cause acute myocardial infarction and an unexpected sudden cardiac death [23].

A young patient presenting acutely with new-onset heart failure or arrhythmias greatly increases the clinical suspicion of the disease. Acute heart failure and arrhythmias caused by GCM do not respond to standard heart failure treatment or aggressive antiarrhythmic drugs, respectively [24,25]. 

Other conditions besides GCM can cause similar symptoms to acute fulminant myocarditis. To distinguish between them, an endomysial biopsy is the most reliable method [24]. It is crucial to diagnose GCM early and differentiate it from other types of myocarditis in order to start immunosuppression therapy promptly. GCM is one of the few causes of fulminant myocarditis where administering immunosuppression in the acute stage has been shown to improve outcomes [23-26].

Subtypes of myocarditis 

Using molecular techniques, mostly reverse transcriptase-polymerase chain reaction (RT-PCR), a variable number of patients with myocarditis and dilated cardiomyopathy (DCM) have viral genomes found in their myocardium. If no infectious organisms are found on an endomyocardial biopsy (EMB) and all other known causes are ruled out, the myocarditis is autoimmune. Autoimmune illnesses, such as systemic lupus erythematosus, can cause extracardiac organ involvement, which can result in autoimmune myocarditis [20-26]. 

Many infectious agents, such as viruses, bacteria, chlamydia, *Rickettsia*, fungi, and protozoa, as well as non-infectious causes like toxins and hypersensitivity reactions, can result in myocarditis. Of these triggers, viral infection has been identified as the leading cause of myocarditis, particularly in children [25].

Immune-mediated myocarditis is also prevalent, with a plethora of causative agents. These include allergens, which are mainly tetanus toxoid, serum sickness, and vaccines. Drugs can cause immune-mediated and toxic myocarditis. Penicillin, cefaclor, amitriptyline, thiazide diuretics, phenytoin, sulfonamides, tetracyclines, lidocaine, and isoniazid are the leading causes of immune-mediated myocarditis, whereas clozapine, interleukin-2, trastuzumab, catecholamines, lithium, ethanol, fluorouracil, cocaine, and anthracyclines are important causes of toxic myocarditis [25].

Alloantigens and autoantigens also cause myocarditis. Alloantigens that cause myocarditis include heart transplant rejection, whereas autoantigens include a number of autoimmune and immune-oriented disorders as well as idiopathic causes. Among the most important idiopathic causes are virus-negative lymphocytic and virus-negative GCM. Wegener’s granulomatosis, sarcoidosis, thyrotoxicosis, myasthenia gravis, polymyositis, scleroderma, inflammatory bowel disease, systemic lupus erythematosus, Churg-Strauss syndrome, rheumatoid arthritis, Kawasaki disease, and insulin-dependent diabetes mellitus are among the leading causes of autoimmune myocarditis [25].

Toxic myocarditis includes agents such as drugs as mentioned above, heavy metals, toxic substances such as scorpion stings, carbon monoxide, bee and wasp stings, phosphorus, arsenic, and inhalants. Heavy metals that can cause myocarditis are copper, iron, and lead. Hormonal diseases such as pheochromocytoma and vitamin deficiency syndromes such as beriberi can also cause myocarditis, and physical agents such as radiation and electric shock are also rare causes [25].

Following the administration of the messenger RNA (mRNA) COVID-19 vaccine to adolescents and young adults, a rising number of serious but uncommon incidents of myocarditis and pericarditis have been documented [26]. Smallpox immunization has been linked to sporadic cases of myocarditis and pericarditis. Myocarditis is one of the more severe vaccine-related side effects. Although myocarditis has been observed after numerous immunizations, the smallpox vaccine has the strongest correlation [27]. While most myocarditis cases completely recover, some individuals experience chronic heart failure or even death. Patients who experience cardiopulmonary symptoms following recent vaccinations should always have vaccine-associated myocarditis on their differential diagnosis list [28]. Another frequent cause of death in those with severe dengue is myocarditis [29]. Apart from these, a few other causes, such as parvovirus B19 and hepatitis B infections, can also lead to myocarditis [30-32].

The severe acute respiratory syndrome coronavirus 2 (SARS-CoV-2) has also been implicated in the development of myocarditis and likely causes myocarditis in humans through a pathway similar to that of other viral pathogens [33]. While SARS-CoV-2 uses the Nsp1 protein to enhance its RNA translation, the Middle East respiratory syndrome coronavirus (MERS-CoV) uses the 4a accessory protein to hinder the creation of cellular stress granules. SARS-CoV-2 may enter cardiac myocytes using those cells' surface angiotensin-converting enzyme 2 (ACE2) receptors and cause immediate cellular damage [33].

Genetic predisposition

The majority of the time, myocarditis is the outcome of an autoimmune reaction to a viral infection. Differences in clinical severity and outcome can be partially attributed to variations in viral genetics as well as the influence of factors such as sex, age, and coexisting health conditions in the host. Because these factors only partially explain the differences in myocarditis risk after exposure to the same injury, clinical and experimental research has sought to identify the role of host genetic vulnerability. Mutations in toll-like receptor 3 (TLR3) make people more vulnerable to enteroviral myocarditis and cardiomyopathy [34]. Interferon-induced transmembrane protein 3 (IFITM3) genetic variation and the severity of influenza infection. Mice that express the human leukocyte antigen (HLA), particularly the HLA-DQ8 molecule from the human major histocompatibility complex (MHC), develop autoimmune myocarditis spontaneously [34]. Other, non-immune methods exist, such as efficient host protein cleavage by viral proteases and cytoskeletal protein deficiencies, such as dystrophin deficiency, increasing the risk of myocarditis-induced cardiomyopathy [34].

A variety of factors, including local or systemic primary autoimmunity, viral infection, HLA and gender predisposition, exposure to concealed antigens, and molecular mimicry, contribute to the development of autoimmune myocarditis [34]. Once the anti-myocardium autoimmune process is initiated, many immune response elements coordinate a prolonged attack against cardiac tissues with specific timing and immunopathogenic traits. Previously undetected recessive abnormalities in non-immunity genes raise the likelihood or severity of viral myocarditis. For instance, dystrophin mutations raise the risk of cardiac coxsackievirus B3 (CVB3) infection. The incidence of potentially harmful mutations in the genes DSP, PKP2, and TNNI3 (encoding, respectively, desmoplakin, plakophilin-2, and troponin I type 3) is elevated in children with acute myocarditis [34].

Pathway enrichment studies in GCM revealed that pathways that were elevated were upgraded for neutrophil degranulation, several cytokine transmission pathways, as well as phagocytosis, were upregulated, whereas cardiac conduction and muscle contraction-related pathways were downregulated [34-42]. The genes that play the most significant roles in innate and adaptive immune responses are tumor necrosis factor alpha-induced protein 2 (TNFAIP2), integrin alpha X (ITGAX), ribonuclease T2 (RNASET2), tumor necrosis factor receptor superfamily member 14 (TNFRSF14), protein tyrosine phosphatase non-receptor type 7 (PTPN7), and HLA-DRA (human leukocyte antigen-DR Alpha). MYL2 (myosin light chain 2), CAMK2B (calcium/calmodulin-dependent protein kinase II Beta), muscular LMNA-interacting protein (MLIP), and A-kinase anchoring protein 6 (AKAP6) were the genes that significantly influenced the contraction of the heart, conductivity, and homeostasis, respectively [42,43].

Several clinical warning signs indicate the need for genetic testing in patients with myocarditis. For instance, intellectual disability may hint at dystrophinopathies or mitochondrial disorders. Neurosensory issues can suggest mitochondrial diseases, while skeletal muscle involvement may imply underlying conditions such as dystrophinopathies, desminopathies, or laminopathies [44]. Clinically, woolly hair and keratoderma indicate possible Carvajal syndrome and pregnancy points towards dilated cardiomyopathy. The recurrence of acute myocarditis and several features on EKG (electrocardiogram), echocardiography, and cardiac magnetic resonance imaging suggest arrhythmogenic cardiomyopathy. On ECG, T negative waves in V1-3 <14y/o or V1-4 >14y/o, Epsilon wave, and, on echocardiography, right ventricular dyskinesia, akinesia, or aneurysm; on cardiac magnetic resonance imaging, adipose infiltration, and diffuse late gadolinium enhancement suggest arrhythmogenic cardiomyopathy [44].

Treatment and management 

*Diagnostic Modalities* 

GCM is an inflammatory heart condition that frequently progresses swiftly and may lead to cardiac failure, arrhythmias, and sudden death. Early detection and treatment are vital to optimizing the prognosis of GCM patients. Several modalities are currently available for diagnosing GCM, including imaging techniques, laboratory tests, and histological examination.

Electrocardiogram (EKG)

The electrocardiogram (EKG) is a valuable tool in the early workup of individuals with suspicions of GCM. However, the EKG results in GCM patients are not specific and vary widely [45]. The most common ECG abnormalities in GCM are sinus tachycardia, atrial fibrillation, ventricular arrhythmias, and conduction anomalies such as left bundle branch block or atrioventricular block. However, it is important to note that these findings can also occur in other forms of heart disease. The sensitivity of EKG in the diagnosis of GCM is reported to be around 50% [46].

Cardiac Biomarkers

The bloodwork in patients suspected of having GCM includes a complete blood count, erythrocyte sedimentation rate, C-reactive protein, and cardiac biomarkers such as troponin and brain natriuretic peptide (BNP). Cardiac biomarkers are valuable for estimating the severity of heart failure and determining the prognosis of GCM patients. The sensitivity and specificity of cardiac troponins in diagnosing GCM are varied, and no consensus exists regarding cutoff values. Troponin and BNP levels are commonly elevated in GCM patients, and the extent of the increase corresponds with the degree of heart failure. However, these tests are not specific for GCM and can also be elevated in other types of heart disease.

Gilotra et al. investigated the association between serum cardiac troponin I levels and the prognosis and diagnosis of GCM [46]. They discovered that individuals with GCM and those with other types of myocarditis or dilated cardiomyopathy did not significantly differ in their troponin I levels [46]. This suggests that cardiac troponin levels are not specific to GCM and cannot be relied upon as a diagnostic marker for this condition. This information aligns with the conclusions of earlier studies that have shown no meaningful relationship between cardiac troponin I and the occurrence of GCM [46-49].

Current research in this area has identified several potential biomarkers for GCM, including myocarditis-associated immune cells, cytokines, and other chemicals, as well as the comparatively recent discovery of miRNAs abundantly expressed in myocarditis [50].

Imaging

The diagnosis of GCM can be assisted by imaging techniques, including echocardiography and cardiac magnetic resonance imaging (MRI).

Echocardiography: An easily accessible and popular non-invasive imaging technique for diagnosing GCM is echocardiography. Patients with GCM may have global or localized anomalies in wall motion, thickening of the ventricular myocardium, and decreased ejection fraction. These results, nevertheless, are general and can also be found in other types of cardiac disease.

Cardiac MRI: In the diagnosis of GCM, cardiac magnetic resonance imaging is more specific and sensitive than echocardiography. However, papers reporting cardiac MRI results in GCM are few, owing to the fact that cardiogenic shock and dangerous arrhythmias often make MRI impractical. Patients with GCM have MRI results that show hyperintense myocardial signals on T2-weighted imaging, late gadolinium enhancement, and reduced myocardial strain. These observations are suggestive of myocardial inflammation and fibrosis, which are hallmarks of GCM. According to reports, the specificity and sensitivity of cardiac MRI in the identification of GCM are approximately 87-88% and 80-93%, respectively [51,52].

Myocardial Biopsy

Myocardial biopsy remains the gold-standard diagnostic modality for GCM [52]. This procedure involves the removal of a small sample of myocardial tissue for microscopic examination. The histopathological findings of EMB in GCM typically show multifocal inflammation with giant cells, myocyte necrosis, and fibrosis [53].

The presence of giant cells in the myocardial biopsy specimen is the hallmark feature of GCM. The giant cells are typically located in the interstitium or around blood vessels and are characterized by their large size, multinucleation, and cytoplasmic granules. Other essential features that suggest a diagnosis of GCM include a diffuse infiltration of inflammatory cells, myocyte necrosis, and interstitial fibrosis. The inflammatory cells infiltrate the myocardium diffusely, and there is often patchy fibrosis [53]. In severe cases, there may be evidence of myocyte damage, including myocyte vacuolization and myocyte dropout. The extent and severity of these histopathological characteristics may vary among GCM patients, and their existence should be analyzed in the context of the patient's clinical condition.

Several studies have demonstrated the diagnostic value of myocardial biopsy for GCM. According to a 2012 investigation, a myocardial biopsy had a diagnostic sensitivity of 95% for 19 of 20 patients with suspected GCM [54]. The sensitivity of myocardial biopsy was reported to be 93% by Kandolin et al. in a separate study [2].

In addition to its diagnostic utility, the myocardial biopsy is also essential for guiding treatment. GCM is a severe form of myocarditis that often requires aggressive immunosuppressive therapy, including corticosteroids, cyclosporine, and mycophenolate mofetil. A myocardial biopsy can help identify patients likely to benefit from these therapies and monitor their response to treatment [55]. Myocardial biopsy has an evolving role in determining the prognosis of GCM. The level of myocardial fibrosis was identified as a distinct predictor of death in research conducted by Ekström et al. on the histological findings of myocardial biopsies in patients with GCM [56]. Therefore, a myocardial biopsy can provide prognostic information that can guide treatment decisions.

Nevertheless, obtaining an endomyocardial biopsy (EMB) for the diagnosis of giant cell myocarditis has several limitations. Firstly, the procedure itself poses risks, particularly in individuals with existing heart problems, and can lead to complications and long-term issues such as tricuspid regurgitation if performed repeatedly. Secondly, it is crucial to have an experienced cardiac pathologist review the EMB results, as giant cell myocarditis can be easily mistaken for cardiac sarcoidosis, emphasizing the need for expert confirmation in suspected cases [56].

Myocardial Gene Profiling

Myocardial gene profiling is a promising modality that may help diagnose GCM. Gene expression analysis can provide information on the underlying molecular mechanisms of GCM and distinguish it from other forms of myocarditis. Studies have shown that GCM is associated with a distinct gene expression signature characterized by the upregulation of genes involved in inflammation and immune activation [57]. However, gene profiling is currently only available in research settings and is not routinely used in clinical practice.

*Diagnostic Differentials* 

The following conditions may exhibit symptoms comparable to GCM:

Cardiac sarcoidosis (CS) refers to cardiac abnormalities associated with sarcoidosis, a condition defined by the abnormal formation of granulomas in numerous organs of the body. Depending on the exact organs affected and the extent of their involvement, the variety and severity of sarcoidosis symptoms vary considerably. In certain instances, only the heart may be affected (cardiac sarcoidosis). Just a limited fraction of patients can be diagnosed with GCM or CS based only on their initial clinical presentation, necessitating a cardiac tissue diagnosis [58]. Early diagnosis of GCM or CS is essential, as individualized immunosuppressive therapy can drastically modify the clinical outcome of these patients [59]. According to a study conducted in Sweden, GCM had a more severe course, a higher level of cardiac biomarkers, and a greater requirement for heart transplantation than cardiac sarcoidosis [60]. The idea that they represent distinct phenotypes of a single disease has been disputed but not conclusively established [61].

Lymphocytic myocarditis is an uncommon disorder in which myocarditis is caused by lymphocyte buildup. Some signs and symptoms include discomfort in the chest, a rapid heart rate, dizziness, lightheadedness, and fatigue, especially with activity or while resting flat. Viruses are typically the cause of lymphocytic myocarditis [62]. In a retrospective analysis, Hu et al. found that patients with fulminant GCM had a considerably worse prognosis than those with fulminant lymphocytic myocarditis. High-sensitivity C-reactive protein (hs-CRP) was also identified as a useful biomarker for distinguishing between the two diseases [63].

Hypersensitivity myocarditis is the general term for myocarditis caused by an allergic reaction to substances such as medications. On the other hand, GCM is not typically related to adverse medication reactions. Symptoms may include chest pain, heart palpitations, tiredness, and dyspnea, particularly during physical effort or when lying flat. Histologically, both disorders exhibit eosinophilic infiltration; however, in the case of hypersensitivity myocarditis (HSM), the infiltration is primarily perivascular [64]. There have been documented cases with histologic characteristics resembling GCM but resulting from a drug reaction [65].

Treatment Options

The current standard of care for GCM involves aggressive immunosuppressive therapy, typically with a combination of corticosteroids and other immunomodulatory drugs [66]. Without immunosuppressive medication, the chance of mortality or cardiac surgery in patients with GCM was formerly 100%, with a median period from symptom onset of three months. Immunosuppression is critical in the evaluation and management of GCM. Immunosuppressive therapy helps reduce the inflammation and structural damage caused by the immune system in GCM patients. Many studies have shown that immunosuppression is useful in treating GCM.

Immunosuppression

In GCM, the major objective of immunosuppressive medication is to avoid the progression of the disease and the necessity for cardiac transplantation. The use of immunosuppressive agents in the management of GCM is based on the theory that GCM is an autoimmune disease, and immunosuppressive therapy can prevent further damage to the myocardium. The most commonly used immunosuppressive agents in the management of GCM are highlighted in Table 1 [67-83].

**Table 1:** Immunosuppressive therapy in the treatment of GCM NA: not applicable; IV: intravenous; DNA: deoxyribonucleic acid; ITP: immune thrombocytopenic purpura; IL: interleukin; UTI: urinary tract infection; WBC: white blood cell; EBV: Epstein-Barr virus; GBM: glomerular basement membrane

Drug	Mechanism of action	Dose	Contraindications	Maintenance therapy	Duration	Adverse effects
MethylPrednisolone	Binds to and activates specific nuclear receptors, altering gene expression and inhibiting proinflammatory cytokine production [68-70]	Pulse therapy: 10 mg/kg up to 1000 mg/day) daily	Hypersensitivity to methylprednisolone, immune thrombocytopenia, systemic sclerosis	NA	3 days	Peptic ulcer, ulcerative esophagitis [69], hyperglycemia [71,72], infection [73], myopathy [69], and glaucoma [74]
Prednisone	Same as methylprednisolone	1 mg/kg/day	Infection, hypopituitarism	Tapered gradually, after 6–8 weeks, the dose is reduced to 5–10 mg/day.	Stop after 1 year or 5 mg/day indefinitely.	Infection [73], hypertension, hyperglycemia [71,72], peptic ulcer [69], Cushing's syndrome [75], osteoporosis [76], cataract [77], and psychosis
Cyclosporine	Inhibits T-cell activation by blocking the synthesis of IL-2.	Variable	Infection [13, 14], impaired renal function, attenuated vaccine	First 1-3 months: 150-300 ng/ml; 4-12 months: 100-150 ng/ml	75-100 ng/ml indefinitely	hypertension, arrhythmias, gynecomastia, hypertrichosis, encephalopathy, and infection [78,79].
Azathioprine	Inhibits DNA synthesis in B and T cells	1.5-2 mg/kg/day	Chronic kidney disease stage 3A-5, pregnancy,	Withhold temporarily if the WBC count < 3,000/ML or decrease by half of the previous value.	1 year	Pancytopenia, malignant lymphoma, acute pancreatitis, infection, and hepatotoxicity
Tacrolimus	Inhibits calcineurin and IL-2 gene transcription [80]	variable	Hypersensitivity [16], polyoxyl 60, hydrogenated castor oil (HCO-60)	1-6 months-10-15 ng/ml	5–10 ng/ml for 1 year or indefinitely	Arrhythmias, abnormal dreams, rash, acute renal failure, and UTI
Rituximab	Anti-CD-20 monoclonal antibody	375 mg/m2/week intravenously for 4 weeks [82]	Heart failure, pregnancy, and live vaccine hypersensitivity	4 months	1 year	Allergic or anaphylactic reactions, infections, lymphopenia, Steven Johnson syndrome, and arrhythmias
Mycophenolate mofetil	Inhibits DNA synthesis in B and T lymphocytes	1.5 gm twice daily	Pregnancy, live vaccine hypersensitivity	The target trough level is 2-5 micrograms/ml	6 months	Leucopenia, dermatological malignancy, lymphoma, progressive multifocal leukoencephalopathy, and vision changes
Antithymocyte globulin	Rapid decrease in T cells	100 mg IV	Thrombocytopenia, leucopenia	Discontinue the drug when WBC< 2000/ml or platelet count <50,000/ml	3 days	Anaphylaxis, serum sickness, cytokine release syndrome, acute thrombosis, and EBV-associated lymphoproliferative malignancies
Alemtuzumab	Targets CD52 antigen on B and T lymphocytes and depletes them.	30 mg IV once, 15 mg IV for 2 days	Stroke, pregnancy, low CD4 count	NA	1-2 days	Hematuria, hypotension, autoimmunity, melanoma, ITP, anti-GBM disease, and lymphoproliferative disorders

Typically, GCM is initially treated with intravenous methylprednisolone at a dose of 10 mg/kg for three days (not exceeding 1,000 mg/day), followed by a gradual taper of prednisone. The prednisone regimen usually begins at 40-60 mg/day and is reduced to 5-10 mg/day after six to eight weeks. Along with this, cyclosporine and either anti-thymocyte globulin (100 mg/day for three days) or alemtuzumab (15 mg/day for two days) are administered. Another potential treatment option involves high-dose corticosteroids in combination with cyclosporine and azathioprine (at a dose of 1.5-2 mg/kg/day) [84].

One retrospective study of patients with GCM found that treatment with a combination of corticosteroids and cyclosporine significantly improved cardiac function and reduced mortality rates compared to patients who did not receive immunosuppression [85]. According to a study by Cooper et al., the transplantation-free survival (TFS) rate for patients who only received corticosteroids was equivalent to that for those who were not given immunosuppressive therapy, suggesting that corticosteroids alone do not significantly prolong survival. The TFS of individuals managed with cyclosporine and other medications increased from three to 12.6 months [86]. In this multicenter, prospective research, the patients treated with cyclosporine and corticosteroids, regardless of the status of muromonab-CD3, had a one-year survival rate of 91%, with one mortality and two patients requiring transplantation within the first month. In this research, it was found that immunosuppression resulted in a substantial decrease in the number of giant cells, locations of lymphocytic myocarditis, eosinophilic infiltration, and necrosis between the initial evaluation and four weeks later, as demonstrated by biopsy samples. The study also assessed the left ventricular ejection fraction (LVEF) before and after immunosuppression. During four weeks of immunosuppression, the mean LVEF did not substantially differ from the baseline value (p = 0.60) [86].

In a second study involving 26 patients from the same multicenter GCM registry, cyclosporine was associated with a decreased likelihood of heart transplantation or mortality. Also, it was indicated that long-term immunosuppression could extend transplantation-free life by >19 years after first diagnosis while maintaining tolerable safety. Immunosuppression discontinuation, or sometimes reduction, is linked with GCM recurrence up to eight years after diagnosis [87]. Recent studies have suggested combining tacrolimus and mycophenolate mofetil may be a more effective treatment option for patients with GCM than cyclosporine and azathioprine [88, 89]. Tacrolimus and mycophenolate mofetil are believed to have synergistic effects in suppressing the immune system, improving outcomes, and reducing morbidity in patients with GCM [90]. Thus, tacrolimus and mycophenolate mofetil are used together with prednisone in some locations to treat GCM. Nevertheless, azathioprine or mycophenolate mofetil combined with tacrolimus or cyclosporine is acceptable.

Despite its rarity, relapse of GCM is relatively prevalent and may be a substantial worry for individuals who have previously been diagnosed with the disorder. Recent studies show that the risk of recurrence of GCM is between 20% and 40% [91]. The exact causes of GCM recurrence are still not entirely understood. However, it has been suggested that the reactivation of viral infections and autoimmune responses may play important roles. Recurrence of GCM has been reported up to eight years after the first diagnosis [87]. For patients with long-term left ventricular (LV) dysfunction, a single immunosuppressive drug is typically prescribed at a low dosage for a minimum of two years and often continuously, although there is no conclusive evidence to support the use of supplementary immunosuppressants beyond the first year.

Treatment including combined immunosuppression and mechanical circulatory support (MCS) was connected to greater overall survival compared to treatment with MCS alone when looking at the patient subgroups with increased survival. Immunosuppression may lessen the intensity of heart failure in these patients by halting the progression of the autoimmune myocardial damage. Due to their less acute heart failure when they arrived, these patients may also have had superior survival rates. In a comprehensive analysis, Patel et al. found that patients who got MCS plus immunosuppression (IS) had significantly greater LVEFs than patients who received MCS alone (28% vs. 17%, p = 0.03) [92]. According to previous research, patients who present with less severe symptomatology or a more gradual course typically have better outcomes than those who do so with acute cardiac compromise [93].

However, it is important to balance the benefits of immunosuppression with potential adverse effects such as the increased risk of infection and cardiovascular toxicities. Therefore, careful monitoring for potential side effects and close collaboration between cardiologists and immunologists are essential to achieving optimal outcomes in GCM patients receiving immunosuppressive therapy.

Mechanical Circulatory Support 

GCM can cause hemodynamic instability as a result of biventricular failure or ventricular arrhythmias. This may necessitate the administration of inotropic therapy or the implementation of temporary mechanical circulatory support. In severe cases, patients may need intra-aortic balloon pump insertion, other types of temporary MCS, or extracorporeal life support if they do not respond to conventional treatments [94,95]. 

Patients who have serious cardiac or respiratory failure may receive temporary cardiac and/or respiratory assistance using extracorporeal membrane oxygenation (ECMO), a form of extracorporeal life support [96]. ECMO can be utilized to support the heart and lungs in cases of GCM, allowing immunosuppressive medication to take effect.

The potential benefits of ECMO in treating GCM have been suggested by a number of case reports and case series [97]. A retrospective study determined that venoarterial ECMO as a rescue modality confers a considerable survival advantage compared to historical cohorts. ECMO was also related to a marked improvement in heart function and decreased inflammatory markers.

ECMO has been used to treat patients with acute fulminant myocarditis-related cardiogenic shock as a bridge to cardiac transplant surgery or to enhance myocardial recovery [97,98]. Throughout the past three decades, venoarterial ECMO has been utilized as the predominant form of ECMO to treat myocarditis, with varied percentages of survival until hospital discharge. When compared to previous cohorts, the application of venoarterial ECMO in children with myocarditis has been observed to have a significant survival benefit [99]. Percutaneous ECMO is a highly efficient type of hemodynamic support in patients with fulminant myocarditis, with a favorable result found in those who recover from inflammatory myocardial injury. The use of an intra-aortic balloon pump (IABP) has been suggested as a possible intervention for GCM. Most studies on IABP therapy have focused on its application in treating cardiogenic shock, refractory left ventricular failure, and coronary revascularization procedures [100]. In general, the evidence indicates that IABP therapy is associated with improved hemodynamics and coronary blood flow. IABP therapy may reduce cardiac afterload and improve diastolic inflow during intra-aortic balloon pumping, thereby providing hemodynamic stabilization and attenuating clinical disturbances of myocardial ischemia [101-103]. 

In controlling GCM, the combination of immunosuppressive medication with MCS has demonstrated good effects [104]. GCM has traditionally been treated with high-dose steroids and other immunosuppressive medications, but the addition of MCS has demonstrated a noteworthy improvement in prognosis for many patients. The combination of immunosuppressive medication with MCS has been demonstrated to increase survival rates and outcomes in GCM patients. Yet, the ideal timing and duration of immunosuppression and MCS in the treatment of GCM are currently being studied. Patients with GCM require close monitoring and tailored treatment programs to achieve the best possible outcomes [105,106]. 

*Cardiac Transplant* 

Despite good medical treatment, numerous patients progress to cardiac transplantation [91]. Myocarditis with giant cells frequently necessitates cardiac transplantation due to increasing heart failure. Heart transplantation remains the only successful therapy in the long term [91]. Despite the risk of allograft recurrence, transplantation is a potential therapy option for GCM that is refractory to conventional treatment [107].

After transplantation, 20% to 25% of patients experience recurrent GCM [108]. While some individuals may have heart failure (HF) or other symptoms, the vast majority are asymptomatic, and GCM is often detected by EMB surveillance. EMB may be used in patients who have just received cardiac transplantation and are having new-onset heart block, ventricular arrhythmias, or a decline in left ventricular systolic function. Given the possibility of recurrence following orthotopic heart transplantation, individuals with GCM who receive transplants are routinely kept on low-dose corticosteroids for life. Lifelong immunosuppression at a low dose appears to be necessary. Full termination of immunosuppressant therapy or an unmonitored decrease in dose has led to the return of GCM and even reports of fatal disease relapse in both native and transplanted hearts up to eight years after initial manifestation [108].

## Conclusions

Giant cell myocarditis (GCM) is a rare but life-threatening disease that requires prompt diagnosis and aggressive treatment. The current standard of care for GCM involves immunosuppressive therapy, which helps to reduce the inflammation and structural damage caused by the immune system in GCM patients. Further research is needed to determine the ideal immunosuppressive regimen for patients with GCM and whether the addition of newer immunomodulatory drugs could provide further benefits.

## References

[1] Mann DL, Zipes DP, Libby P, Bonow RO (2014). Braunwald’s Heart Disease: A Textbook of Cardiovascular Medicine. Braunwald’s Heart Disease: A Textbook of Cardiovascular Medicine.

[2] Kandolin R, Lehtonen J, Salmenkivi K, Räisänen-Sokolowski A, Lommi J, Kupari M (2013). Diagnosis, treatment, and outcome of giant-cell myocarditis in the era of combined immunosuppression. Circ Heart Fail.

[3] Cooper LT Jr, ElAmm C (2012). Giant cell myocarditis. Diagnosis and treatment. Herz.

[4] Caforio AL, Pankuweit S, Arbustini E (2013). Current state of knowledge on aetiology, diagnosis, management, and therapy of myocarditis: a position statement of the European Society of Cardiology Working Group on Myocardial and Pericardial Diseases. Eur Heart J.

[5] Golpour A, Patriki D, Hanson PJ, McManus B, Heidecker B (2021). Epidemiological impact of myocarditis. J Clin Med.

[6] Kang M, Chippa V, An J (2023). Viral Myocarditis. https://www.ncbi.nlm.nih.gov/books/NBK459259/.

[7] Davidoff R, Palacios I, Southern J, Fallon JT, Newell J, Dec GW (1991). Giant cell versus lymphocytic myocarditis. A comparison of their clinical features and long-term outcomes. Circulation.

[8] (2023). Giant cell myocarditis. https://my.clevelandclinic.org/health/diseases/23526-giant-cell-myocarditis..

[9] Low Wang CC, Hess CN, Hiatt WR, Goldfine AB (2016). Clinical update: cardiovascular disease in diabetes mellitus: atherosclerotic cardiovascular disease and heart failure in type 2 diabetes mellitus - mechanisms, management, and clinical considerations. Circulation.

[10] Cooper LT Jr, Berry GJ, Shabetai R (1997). Idiopathic giant-cell myocarditis--natural history and treatment. Multicenter Giant Cell Myocarditis Study Group Investigators. N Engl J Med.

[11] Kytö V, Sipilä J, Rautava P (2013). The effects of gender and age on occurrence of clinically suspected myocarditis in adulthood. Heart.

[12] Fairweather D, Cooper LT Jr, Blauwet LA (2013). Sex and gender differences in myocarditis and dilated cardiomyopathy. Curr Probl Cardiol.

[13] Leone O, Pieroni M, Rapezzi C, Olivotto I (2019). The spectrum of myocarditis: from pathology to the clinics. Virchows Arch.

[14] Rosenstein ED, Zucker MJ, Kramer N (2000). Giant cell myocarditis: most fatal of autoimmune diseases. Semin Arthritis Rheum.

[15] Xu J, Brooks EG (2016). Giant cell myocarditis: a brief review. Arch Pathol Lab Med.

[16] Shields RC, Tazelaar HD, Berry GJ, Cooper LT Jr (2002). The role of right ventricular endomyocardial biopsy for idiopathic giant cell myocarditis. J Card Fail.

[17] Berthelot-Richer M, O'Connor K, Bernier M, Trahan S, Couture C, Dubois M, Sénéchal M (2014). When should we consider the diagnosis of giant cell myocarditis? Revisiting "classic" echocardiographic and clinical features of this rare pathology. Exp Clin Transplant.

[18] Mirabel M, Callon D, Bruneval P (2020). Late-onset giant cell myocarditis due to enterovirus during treatment with immune checkpoint inhibitors. JACC CardioOncol.

[19] Reuben A, Petaccia de Macedo M, McQuade J (2017). Comparative immunologic characterization of autoimmune giant cell myocarditis with ipilimumab. Oncoimmunology.

[20] Sumi M, Kitahara M, Shishido T (2020). Myocarditis with advanced atrioventricular block after allogeneic stem cell transplantation: a case report and literature review. Intern Med.

[21] Ekström K, Lehtonen J, Kandolin R, Räisänen-Sokolowski A, Salmenkivi K, Kupari M (2016). Incidence, risk factors, and outcome of life-threatening ventricular arrhythmias in giant cell myocarditis. Circ Arrhythm Electrophysiol.

[22] Batra AS, Lewis AB (2001). Acute myocarditis. Curr Opin Pediatr.

[23] Blauwet LA, Cooper LT (2013). Idiopathic giant cell myocarditis and cardiac sarcoidosis. Heart Fail Rev.

[24] Ghaly M, Schiliro D, Stepczynski J (2020). Giant cell myocarditis: a time sensitive distant diagnosis. Cureus.

[25] Caforio AL, Marcolongo R, Jahns R, Fu M, Felix SB, Iliceto S (2013). Immune-mediated and autoimmune myocarditis: clinical presentation, diagnosis and management. Heart Fail Rev.

[26] Li M, Yuan J, Lv G, Brown J, Jiang X, Lu ZK (2021). Myocarditis and pericarditis following COVID-19 vaccination: inequalities in age and vaccine types. J Pers Med.

[27] Keinath K, Church T, Kurth B, Hulten E (2018). Myocarditis secondary to smallpox vaccination. BMJ Case Rep.

[28] Li Y, Hu Z, Huang Y (2016). Characterization of the myocarditis during the worst outbreak of dengue infection in China. Medicine (Baltimore).

[29] Molina KM, Garcia X, Denfield SW (2013). Parvovirus B19 myocarditis causes significant morbidity and mortality in children. Pediatr Cardiol.

[30] Sengupta P, Biswas S, Roy T (2019). Hepatitis E-induced acute myocarditis in an elderly woman. Case Rep Gastroenterol.

[31] Chen Q, Huang DS, Zhang LW, Li YQ, Wang HW, Liu HB (2018). Fatal myocarditis and rhabdomyolysis induced by nivolumab during the treatment of type B3 thymoma. Clin Toxicol (Phila).

[32] Agdamag AC, Edmiston JB, Charpentier V (2020). Update on COVID-19 myocarditis. Medicina (Kaunas).

[33] Bracamonte-Baran W, Čiháková D (2017). Cardiac autoimmunity: myocarditis. The Immunology of Cardiovascular Homeostasis and Pathology. Advances in Experimental Medicine and Biology, Vol 1003.

[34] Chapman SJ, Hill AV (2012). Human genetic susceptibility to infectious disease. Nat Rev Genet.

[35] Arbustini E, Narula N, Giuliani L, Di Toro A (2020). Genetic basis of myocarditis: myth or reality?. Myocarditis.

[36] Maisch B (2019). Cardio-immunology of myocarditis: focus on immune mechanisms and treatment options. Front Cardiovasc Med.

[37] Li HS, Ligons DL, Rose NR (2008). Genetic complexity of autoimmune myocarditis. Autoimmun Rev.

[38] Tschöpe C, Ammirati E, Bozkurt B (2021). Myocarditis and inflammatory cardiomyopathy: current evidence and future directions. Nat Rev Cardiol.

[39] Rose NR (2011). Critical cytokine pathways to cardiac inflammation. J Interferon Cytokine Res.

[40] Belkaya S, Kontorovich AR, Byun M (2017). Autosomal recessive cardiomyopathy presenting as acute myocarditis. J Am Coll Cardiol.

[41] Fung G, Luo H, Qiu Y, Yang D, McManus B (2016). Myocarditis. Circ Res.

[42] Fang M, Zhang A, Du Y (2022). TRIM18 is a critical regulator of viral myocarditis and organ inflammation. J Biomed Sci.

[43] Baggio C, Gagno G, Porcari A (2021). Myocarditis: which role for genetics?. Curr Cardiol Rep.

[44] Nordenswan HK, Lehtonen J, Ekström K (2021). Manifestations and outcome of cardiac sarcoidosis and idiopathic giant cell myocarditis by 25-year nationwide cohorts. J Am Heart Assoc.

[45] Buttà C, Zappia L, Laterra G, Roberto M (2020). Diagnostic and prognostic role of electrocardiogram in acute myocarditis: a comprehensive review. Ann Noninvasive Electrocardiol.

[46] Gilotra NA, Minkove N, Bennett MK (2016). Lack of relationship between serum cardiac troponin I level and giant cell myocarditis diagnosis and outcomes. J Card Fail.

[47] Cooper LT Jr, Onuma OK, Sagar S, Oberg AL, Mahoney DW, Asmann YW, Liu P (2010). Genomic and proteomic analysis of myocarditis and dilated cardiomyopathy. Heart Fail Clin.

[48] Fender EA, Killu AM, Hodge DO (2015). Abstract 10903: extraction outcomes in patients with congenital heart disease. Circ.

[49] Suresh A, Martens P, Tang WH (2022). Biomarkers for Myocarditis and Inflammatory cardiomyopathy. Curr Heart Fail Rep.

[50] Polte CL, Bobbio E, Bollano E (2022). Cardiovascular magnetic resonance in myocarditis. Diagnostics (Basel).

[51] Akita T, Mori S, Onishi A (2019). Successful triple combination immunosuppressive therapy with prednisolone, cyclosporine, and mycophenolate mofetil to treat recurrent giant cell myocarditis. Intern Med.

[52] Liu S, Zheng L, Shen L (2021). Clinical identification and characteristic analysis of giant cell myocarditis in 12 cases. Front Cardiovasc Med.

[53] Sun Y, Zhao H, Song L, Wang Q, Chu Y, Huang J, Hu S (2015). Histological and ultrastructural features of giant cell myocarditis: report of 3 cases [Article in Chinese]. Zhonghua Bing Li Xue Za Zhi.

[54] Leone O, Veinot JP, Angelini A (2012). 2011 consensus statement on endomyocardial biopsy from the Association for European Cardiovascular Pathology and the Society for Cardiovascular Pathology. Cardiovasc Pathol.

[55] Kalra A, Kneeland R, Samara MA, Cooper LT Jr (2014). The changing role for endomyocardial biopsy in the diagnosis of giant-cell myocarditis. Cardiol Ther.

[56] Ekström K, Lehtonen J, Kandolin R, Räisänen-Sokolowski A, Salmenkivi K, Kupari M (2016). Long-term outcome and its predictors in giant cell myocarditis. Eur J Heart Fail.

[57] Lassner D, Kühl U, Siegismund CS (2014). Improved diagnosis of idiopathic giant cell myocarditis and cardiac sarcoidosis by myocardial gene expression profiling. Eur Heart J.

[58] Okura Y, Dec GW, Hare JM (2003). A clinical and histopathologic comparison of cardiac sarcoidosis and idiopathic giant cell myocarditis. J Am Coll Cardiol.

[59] Funaki T, Saji M, Murai T, Higuchi R, Nanasato M, Isobe M (2022). Combination immunosuppressive therapy for giant cell myocarditis. Intern Med.

[60] Bobbio E, Hjalmarsson C, Björkenstam M (2022). Diagnosis, management, and outcome of cardiac sarcoidosis and giant cell myocarditis: a Swedish single center experience. BMC Cardiovasc Disord.

[61] Ekström K, Räisänen-Sokolowski A, Lehtonen J, Nordenswan HK, Mäyränpää MI, Kupari M (2020). Idiopathic giant cell myocarditis or cardiac sarcoidosis? A retrospective audit of a nationwide case series. ESC Heart Fail.

[62] Ganji M, Ruiz-Morales J, Ibrahim S (2018). Acute lymphocytic myocarditis. J Geriatr Cardiol.

[63] Hu Y, Ren J, Dong X (2021). Fulminant giant cell myocarditis vs. lymphocytic myocarditis:a comparison of their clinical characteristics, treatments, and outcomes. Front Cardiovasc Med.

[64] Gonzalez YO, Cooper LT (2020). Giant cell and hypersensitivity myocarditis. Myocarditis.

[65] Daniels PR, Berry GJ, Tazelaar HD, Cooper LT (2000). Giant cell myocarditis as a manifestation of drug hypersensitivity. Cardiovasc Pathol.

[66] Takei Y, Ejima Y, Toyama H, Takei K, Ota T, Yamauchi M (2016). A case of a giant cell myocarditis that developed massive left ventricular thrombus during percutaneous cardiopulmonary support. JA Clin Rep.

[67] Ericson-Neilsen W, Kaye AD (2014). Steroids: pharmacology, complications, and practice delivery issues. Ochsner J.

[68] Liu D, Ahmet A, Ward L (2013). A practical guide to the monitoring and management of the complications of systemic corticosteroid therapy. Allergy Asthma Clin Immunol.

[69] Streeten DH (1975). Corticosteroid therapy. I. Pharmacological properties and principles of corticosteroid use. JAMA.

[70] Baldwin D, Apel J (2013). Management of hyperglycemia in hospitalized patients with renal insufficiency or steroid-induced diabetes. Curr Diab Rep.

[71] Tamez-Pérez HE, Quintanilla-Flores DL, Rodríguez-Gutiérrez R, González-González JG, Tamez-Peña AL (2015). Steroid hyperglycemia: Prevalence, early detection and therapeutic recommendations: a narrative review. World J Diabetes.

[72] Youssef J, Novosad SA, Winthrop KL (2016). Infection risk and safety of corticosteroid use. Rheum Dis Clin North Am.

[73] Phulke S, Kaushik S, Kaur S, Pandav SS (2017). Steroid-induced glaucoma: an avoidable irreversible blindness. J Curr Glaucoma Pract.

[74] Pivonello R, Isidori AM, De Martino MC, Newell-Price J, Biller BM, Colao A (2016). Complications of Cushing's syndrome: state of the art. Lancet Diabetes Endocrinol.

[75] Berris KK, Repp AL, Kleerekoper M (2007). Glucocorticoid-induced osteoporosis. Curr Opin Endocrinol Diabetes Obes.

[76] Dietrich J, Rao K, Pastorino S, Kesari S (2011). Corticosteroids in brain cancer patients: benefits and pitfalls. Expert Rev Clin Pharmacol.

[77] Pal P, Giri PP, Sinha R (2019). Cyclosporine in resistant systemic arthritis: a cheaper alternative to biologics. Indian J Pediatr.

[78] Shin HS, Grgic I, Chandraker A (2019). Novel targets of immunosuppression in transplantation. Clin Lab Med.

[79] Zhang S, Kodama M, Hanawa H, Izumi T, Shibata A, Masani F (1993). Effects of cyclosporine, prednisolone and aspirin on rat autoimmune giant cell myocarditis. J Am Coll Cardiol.

[80] Nicolai S, Bunyavanich S (2012). Hypersensitivity reaction to intravenous but not oral tacrolimus. Transplantation.

[81] Toscano G, Tartaro P, Fedrigo M, Angelini A, Marcolongo R (2014). Rituximab in recurrent idiopathic giant cell myocarditis after heart transplantation: a potential therapeutic approach. Transpl Int.

[82] Amiri A, Houshmand G, Taghavi S, Kamali M, Faraji M, Naderi N (2022). Giant cell myocarditis following COVID-19 successfully treated by immunosuppressive therapy. Clin Case Rep.

[83] Shangyu L, Yan Y (2020). Distribution of pathological features and inflammatory cells in patients with giant cell myocarditis. Circ Res.

[84] Farinha IT, Miranda JO (2016). Myocarditis in paediatric patients: unveiling the progression to dilated cardiomyopathy and heart failure. J Cardiovasc Dev Dis.

[85] Davies MJ, Pomerance A, Teare RD (1975). Idiopathic giant cell myocarditis--a distinctive clinico-pathological entity. Br Heart J.

[86] Cooper LT Jr, Hare JM, Tazelaar HD (2008). Usefulness of immunosuppression for giant cell myocarditis. Am J Cardiol.

[87] Maleszewski JJ, Orellana VM, Hodge DO, Kuhl U, Schultheiss HP, Cooper LT (2015). Long-term risk of recurrence, morbidity and mortality in giant cell myocarditis. Am J Cardiol.

[88] Tymińska A, Ozierański K, Caforio AL (2021). Myocarditis and inflammatory cardiomyopathy in 2021: an update. Pol Arch Intern Med.

[89] Kociol RD, Cooper LT, Fang JC (2020). Recognition and initial management of fulminant myocarditis: a scientific statement from the American Heart Association. Circulation.

[90] Kim JH, Han N, Kim MG (2018). Increased exposure of tacrolimus by co-administered mycophenolate mofetil: population pharmacokinetic analysis in healthy volunteers.. Sci Rep.

[91] Scott RL, Ratliff NB, Starling RC, Young JB (2001). Recurrence of giant cell myocarditis in cardiac allograft. J Heart Lung Transplant.

[92] Patel PM, Saxena A, Wood CT (2020). Outcomes of mechanical circulatory support for giant cell myocarditis: a systematic review.. J Clin Med.

[93] Montero S, Aissaoui N, Tadié JM (2018). Fulminant giant-cell myocarditis on mechanical circulatory support: management and outcomes of a French multicentre cohort. Int J Cardiol.

[94] Goulbourne CA, Sharma N, Cecchini MJ, Bloom M, Singh A, Skopicki H, Pyo R (2021). Giant cell, giant problems: a case of giant cell myocarditis complicated by cardiogenic shock and refractory ventricular tachycardia managed with a mechanical circulatory support device. Cardiovasc Revasc Med.

[95] Makdisi G, Wang IW (2015). Extra corporeal membrane oxygenation (ECMO) review of a lifesaving technology. J Thorac Dis.

[96] Ripoll JG, Ratzlaff RA, Menke DM, Olave MC, Maleszewski JJ, Díaz-Gómez JL (2016). Hemodynamic transesophageal echocardiography-guided venous-arterial extracorporeal membrane oxygenation support in a case of giant cell myocarditis. Case Rep Crit Care.

[97] Ankersmit HJ, Ullrich R, Moser B (2006). Recovery from giant cell myocarditis with ECMO support and utilisation of polyclonal antithymocyte globulin: a case report. Thorac Cardiovasc Surg.

[98] Gutierrez ME, Anders M, Guffey D (2023). Extracorporeal membrane oxygenation cannulation timing in the pediatric myocarditis population: An exploratory analysis from the Extracorporeal Life Support Organization Registry. Crit Care Explor.

[99] Ahmar W, Leet A, Morton J (2007). Diagnostic dilemmas and management of fulminant myocarditis. Anaesth Intensive Care.

[100] Colyer WR Jr, Moore JA, Burket MW, Cooper CJ (1997). Intraaortic balloon pump insertion after percutaneous revascularization in patients with severe peripheral vascular disease. Cathet Cardiovasc Diagn.

[101] Parissis H, Graham V, Lampridis S, Lau M, Hooks G, Mhandu PC (2016). IABP: history-evolution-pathophysiology-indications: what we need to know. J Cardiothorac Surg.

[102] O'Connor CM, Rogers JG (2012). Evidence for overturning the guidelines in cardiogenic shock. N Engl J Med.

[103] Luo M, Zhou J, Qiu C (2022). Outcome of extracorporeal membrane oxygenation combined with intra-aortic balloon pump hemodynamic support during the percutaneous coronary intervention process for patients with cardiac shock complicating acute myocardial infarction. J Healthc Eng.

[104] Nakajima-Doi S, Mochizuki H, Iwasaki K (2020). Mechanical circulatory support combined with immunosuppression for the treatment of giant cell myocarditis - a single-center experience in Japan. Circ J.

[105] Fallon JM, Parker AM, Dunn SP, Kennedy JL (2020). A giant mystery in giant cell myocarditis: navigating diagnosis, immunosuppression, and mechanical circulatory support. ESC Heart Fail.

[106] Bobbio E, Björkenstam M, Nwaru BI (2022). Short- and long-term outcomes after heart transplantation in cardiac sarcoidosis and giant-cell myocarditis: a systematic review and meta-analysis. Clin Res Cardiol.

[107] Toscano G, Tartaro P, Fedrigo M, Angelini A, Marcolongo R (2014). Rituximab in recurrent idiopathic giant cell myocarditis after heart transplantation: a potential therapeutic approach. Transpl Int.

[108] Das BB, Recto M, Johnsrude C (2006). Cardiac transplantation for pediatric giant cell myocarditis. J Heart Lung Transplant.

